# High median nerve lesion secondary to severe giant cell arteritis

**DOI:** 10.1093/rap/rkac019

**Published:** 2022-08-16

**Authors:** Kundan Iqbal, James Miller, Ming Lai, Ben Thompson

**Affiliations:** Translational and Clinical Research, Faculty of Medical Sciences; Department of Rheumatology; Department of Neurology; Department of Neurophysiology, Newcastle upon Tyne Hospitals NHS Foundation Trust, Newcastle upon Tyne, UK; Translational and Clinical Research, Faculty of Medical Sciences; Department of Rheumatology

Key message

- In non-specifically unwell elderly or suspected coronavirus disease 2019 patients, consider GCA and potential peripheral nerve lesions.

Dear Editor, GCA is a systemic vasculitis of medium to large arteries. The aetiology remains incompletely understood but is thought to involve genetic and environmental factors triggering an autoimmune inflammatory response to endothelial injury. Cranial nerve lesions are frequently documented secondary to GCA, but peripheral nerves are rarely involved. We present an unusual case of a GCA-associated isolated high median nerve palsy.

A white British female in her 70s, residing in Spain, with a past medical history of essential hypertension and mild bronchiectasis, presented after 3 weeks of severe frontotemporal headaches, jaw claudication and visual symptoms. She had severe tongue pain, odynophagia and dysarthria, and the anterior part of her tongue appeared swollen and discoloured with pale patches. She also had constitutional symptoms of fatigue, weakness and weight loss. On the way to the hospital, she noted right hand paresis and altered sensation. She had no previous right arm impairment.

On admission to hospital in Spain, her acute-phase reactants were elevated (CRP 290 mg/l; D-dimer 1500 ng/ml; leucocytes 12.1 × 10^9^/l; ferritin 742 µg/l; ESR unavailable). CT head did not demonstrate acute pathology. Chest X-ray demonstrated bibasal infiltrates, possibly secondary to pre-existing bronchiectasis. Severe acute respiratory syndrome coronavirus 2 (SARS-CoV-2) PCR was negative. She was commenced on i.v. antibiotics for suspected community-acquired pneumonia.

Three days after admission, she developed acute bilateral anterior ischaemic optic neuropathy. She was commenced immediately on high-dose methylprednisolone (1 g i.v. daily for 3 days), resulting in a significant decrease in inflammatory markers (CRP decreased 290 mg/l to 16 mg/l 2 days after the final pulse).

Subsequent temporal artery US and biopsy confirmed GCA. Fluorodeoxyglucose PET-CT showed no evidence of a diffuse large vessel vasculitis. Her condition was complicated by tongue infarction attributable to ischaemia, with subsequent autoamputation of the anterior two-thirds.

Given the aggressive disease course, it was decided to commence tocilizumab (162 mg s.c. weekly) 12 days after the diagnosis of GCA, which was well tolerated and effective (CRP <0 mg/l 11 days after treatment commenced). She continued to receive prednisolone in addition (1 mg/kg p.o. daily, commenced after initial i.v. methylprednisolone and tapered by 5 mg every week). She also required percutaneous endoscopic gastrostomy feeding and careful multidisciplinary rehabilitation.

Eight weeks after presentation, she returned to the UK for ongoing inpatient care. At the time of transfer, she was taking tocilizumab (162 mg s.c. weekly) and prednisolone (30 mg p.o. daily) as instituted in Spain.

During her admission in the UK, she described ongoing right arm paresis and altered sensation, at the same severity since initial onset. On examination, she had thinning of the right thenar eminence and hypersensitivity in the distribution of the median nerve. She had weakness of abductor pollicis brevis (MRC grade 2), opponens pollicis (grade 4), pronation (grade 4) and long finger flexion of the second digit (grade 1–2), with no flexion of the first digit DIP joint. Finger abduction and extension were preserved.

Upper limb nerve conduction studies demonstrated a reduced median motor response and absent sensory responses, and needle EMG demonstrated denervation in flexor carpi radialis. These findings were consistent with an isolated right high median nerve lesion proximal to the branch point of flexor carpi radialis. There was no evidence of a more diffuse neuropathy. MRI head and CT head angiogram excluded a central lesion. The timing of her symptoms suggested that the nerve lesion was secondary to GCA.

After a further 5 weeks as an inpatient, she was discharged home on tocilizumab and tapering CSs (prednisolone 10 mg p.o. daily, reducing by 1 mg every 14 days). Given that tocilizumab is recommended for a maximum duration of 12 months by the UK National Institute for Health and Care Excellence (NICE), she was also commenced on MTX (25 mg p.o. weekly) 2 months after discharge as maintenance therapy in anticipation of discontinuing the tocilizumab. Inflammatory markers at 2-month follow-up were normal (CRP <5 mg/l).

Six months after discharge, inflammatory markers remained normal (CRP <5 mg/l), and there had been a substantial improvement in right hand strength and tongue mobility. Further gradual further improvement is predicted over the next 1–2 years, with any residual disability expected to be functionally mild.

This complex case highlights that, although rare, peripheral nerve lesions can occur in GCA. A literature search identified a modest number of case reports of brachial plexus or lower cervical nerve lesions [1, 2]. Neurological manifestations in GCA are attributed to vasculitis of the vasa nervorum or extension of inflammation from arteries to contiguous nerves [1, 2]. The restriction of clinical involvement to the median nerve alone would appear to favour the former mechanism. Functional recovery of neurological deficits is typically partial, at best.

This case also highlights the difficulty faced by clinicians in recognising GCA and the importance of urgent treatment with glucocorticoids. The patient developed GCA during the ongoing coronavirus disease 2019 (COVID-19) pandemic when Spain was the second-worst affected European country (5% seroprevalence; 95% CI 4.7, 5.4) [3]. The partial overlap in symptoms between GCA and COVID-19 (including headache, fatigue, malaise and elevated acute-phase reactants) can cause diagnostic confusion and treatment delay [4, 5]. GCA should be considered in the differential diagnosis of older patients with suspected COVID-19 or in the non-specifically unwell elderly patient.

## Data Availability

Data are available upon reasonable request by any qualified researchers who engage in rigorous, independent scientific research, and will be provided following review and approval of a research proposal and Statistical Analysis Plan (SAP) and execution of a Data Sharing Agreement (DSA). All data relevant to the study are included in the article.
